# The effect of oral motor intervention with different initiation times to improve feeding outcomes in preterm infants: protocol for a single-blind, randomized controlled trial

**DOI:** 10.1186/s13063-024-08131-8

**Published:** 2024-05-07

**Authors:** Yingxin Li, Yanlin Hu, Yuan Li, Xia Li, Xi Huang, Zeyao Shi, Ru Yang, Xiujuan Zhang, Qiong Chen

**Affiliations:** 1 Department of Neonatology Nursing, West China Second University Hospital, Sichuan University, Sichuan, China; 2 Key Laboratory of Birth Defects and Related Diseases of Women and Children, Ministry of Education, No. 20, Section 3, South Renmin Road, Chengdu, Sichuan 610041 China

**Keywords:** Preterm infant, Feeding and eating disorders, Oral motor intervention

## Abstract

**Background:**

Premature infants commonly encounter difficulties with oral feeding, a complication that extends hospital stays, affects infants’ quality of life, and imposes substantial burdens on families and society. Enhancing preterm infants’ oral feeding skills and facilitating their transition from parenteral or nasal feeding to full oral feeding pose challenges for neonatal intensive care unit (NICU) healthcare professionals. Research indicates that oral motor interventions (OMIs) can enhance preterm infants’ oral feeding capabilities and expedite the transition from feeding initiation to full oral feeding. Nonetheless, the most suitable timing for commencing these interventions remains uncertain.

**Methods:**

This is a single-blind, randomized controlled trial. Preterm with a gestational age between 29^+0^ to 34^+6^ weeks will be eligible for the study. These infants will be randomized and allocated to one of two groups, both of which will receive the OMIs. The intervention commences once the infant begins milk intake during the early OMIs. Additionally, in the late OMIs group, the intervention will initiate 48 h after discontinuing nasal continuous positive airway pressure.

**Discussion:**

OMIs encompass non-nutritive sucking and artificial oral stimulation techniques. These techniques target the lips, jaw, muscles, or tongue of premature infants, aiming to facilitate the shift from tube feeding to oral feeding. The primary objective is to determine the ideal intervention timing that fosters the development of oral feeding skills and ensures a seamless transition from parenteral or nasal feeding to full oral feeding among preterm infants. Furthermore, this study might yield insights into the long-term effects of OMIs on the growth and neurodevelopmental outcomes of preterm infants. Such insights could bear substantial significance for the quality of survival among preterm infants and the societal burden imposed by preterm birth.

**Trial registration:**

chictr.org.cn ChiCTR2300076721. Registered on October 17, 2023.

## Administrative information

**Table:** 

Title {1}	The effect of oral motor intervention with different initiation times to improve feeding outcomes in preterm infants: protocol for a single-blind, randomized controlled trial
Trial registration {2a and 2b}.	chictr.org.cn ChiCTR2300076721 date of registration: October 17, 2023
Protocol version {3}	version 1
Funding {4}	This study was supported by the nursing department, West China Second University Hospital, Sichuan University, Sichuan, China [grant number: HLBKJ202023].
Author details {5a}	Yingxin Li, Yanlin Hu, Yuan Li, Xia Li, Xi Huang, Zeyao Shi, Ru Yang, Xiujuan Zhang, Qiong Chen, West China Second University Hospital, Sichuan University, Sichuan, China
Name and contact information for the trial sponsor {5b}	Yingxin Li, yingxin-li@scu.edu.cn West China Second University Hospital, Sichuan University, Sichuan, China
Role of sponsor {5c}	design and conduct of the study

## Introduction

### Background and rationale {6a}

According to the latest data published by Lancet, the global scene records approximately 13.4 million premature births annually, with the incidence rates varying from 6.8 to 16.2% across 103 countries [1]. Within this context, China accounts for 753,000 of these preterm infants, ranking it as the fourth highest in the world in terms of the number of infants born prematurely [1, 2]. Amid the challenging backdrop of NICUs, one glaring hurdle faced by preterm infants is the acquisition of oral feeding skills, a pivotal facet for their growth, and survival [3, 4]. However, a significant proportion of preterm infants encounter struggles in mastering oral feeding, with an incidence rate reaching up to 40% [5, 6]. This struggle primarily presents issues with sucking, swallowing, and even breathing coordination. These feeding difficulties not only extend hospitalization periods but also impact the quality of life for the infants, placing a significant strain on families and society [5, 6].

Mitigating these feeding challenges, improving oral feeding competencies among preterm infants, and facilitating their seamless shift from parenteral nutrition and nasal feeding to full oral feeding constitute shared concerns and hurdles for NICU healthcare professionals. At the crux of this challenge may lie early oral motor interventions (OMIs), presenting a potential solution. Recent studies reveal that OMIs can improve the oral motor function and oral feeding performance of preterm infants.

The concept of OMIs, as first introduced by the American scholars Fucile et al., encompasses interventions that involve non-nutritive sucking, manual oral stimulation, and similar techniques targeting the lips, jaw, tongue, and muscles of preterm infants [7, 8]. The aim is to aid their transition from nasal to oral feeding. A pivotal study by Arora et al. utilizing the Premature Infant Oral Motor Intervention (PIOMI) as pre-feeding stimulation showed that preterm infant undergoing PIOMI achieved full independent spoon-feeding more rapidly, exhibited greater weight gain, and had lower rates of oral aversion than those in the control group [9].

Additional support for the efficacy of OMIs comes from Aguilar-Rodríguez et al.,  who found that such interventions accelerate the progression to full oral feeding and shorten hospital stays [10]. Systematic and meta-analysis reinforce this conclusion, establishing OMIs as an efficacious strategy for bolstering oral feeding in preterm infants [11–14]. Lv et al.’s work underscores the positive effect of early motor interventions on oral feeding ability of preterm infants [15]. Similarly, Wu et al. concluded that oral massage not only improves sucking efficiency but also diminishes the risk of adverse events such as infections and apnea [16]. Wang et al. further validate that OMIs improved feeding performance in preterm infants and did not increase the occurrence of complications associated with prematurity [17]. Thus, integrating OMIs in NICU presents several significant clinical benefits, including: (1) improving oral feeding skills and reducing the transition period from tube feeding or parenteral nutrition to full oral feeding, thereby increasing feeding efficiency; (2) improving the quality of survival by reducing hospitalization durations; and (3) providing early sensory stimulation to support neurobehavioral development in preterm infants.

Despite these advancements, establishing a standardized timing for initiating OMIs remains elusive. Diverse studies advocate different start times, ranging from the infant’s first day following admission [17] to 48 h after ceasing non-invasive ventilation [18, 19], or at the onset of feeding [15]. The cumulative effects of these divergent starting points remain nebulous, compounded by the lack of long-term follow-up research that would elucidate their extended impacts. Consequently, the primary objective of this study is to compare the effects of different initiation times for OMIs on the feeding progress among preterm infants. Simultaneously, it endeavors to evaluate the long-term outcomes of OMIs on both feeding efficiency and neurodevelopment outcomes in preterm infants.

## Objectives {7}

(i) Feeding outcomes: To determine the influence of differing initiation times of OMIs on feeding outcomes among preterm infants.
(ii) Long-term growth and development: To examine the effects of OMIs with varying initiation times on extended growth and development trajectory of preterm infants.
(iii) Neurodevelopmental outcomes: To explore the effects of OMIs with different initiation times on neurodevelopmental outcomes in preterm infants.

## Trial design {8}

**Fig. 1:**
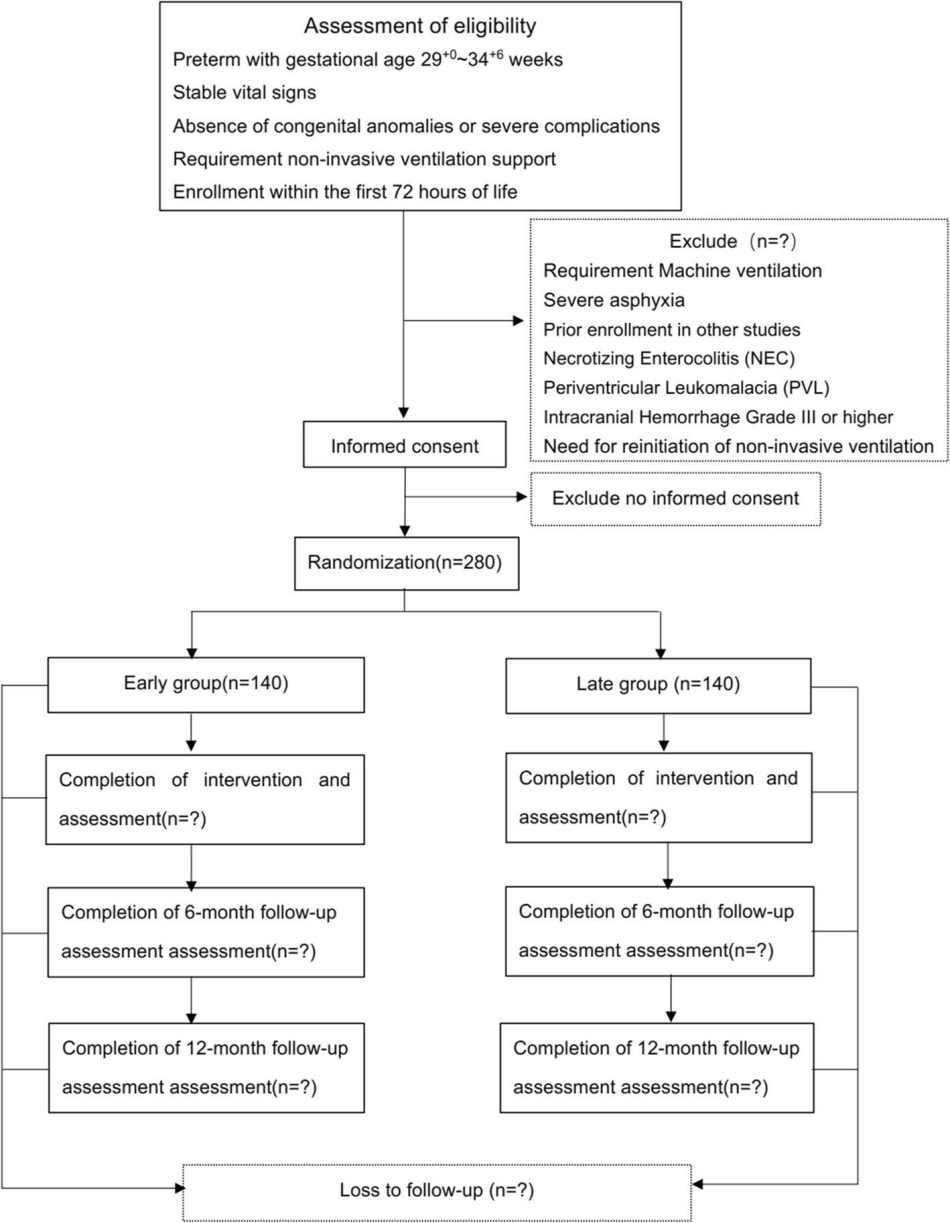
flow chart

This study employs a single-blind, randomized, and controlled trial design, assigning preterm infants to one of two groups: early OMIs or late OMIs. The flow chart is presented in Fig. 1.

## Methods: participants, interventions, and outcomes

### Study setting {9}

This research will take place at the NICU which is a 307-bed tertiary-level referral unit of the West China Second University Hospital, Sichuan University, Chengdu, Sichuan Province, China. This study will be performed during the period from October 20, 2023, to October 20, 2025.

### Eligibility criteria {10}

Inclusion criteria

1. Diagnosis as preterm, with a birth weight of less than 2500 g, and gestational age ranging from 29^+0^ to 34^+6^ weeks.
2. Stability of vital signs.
3. Absence of major congenital anomalies, such as congenital heart disease, or congenital digestive tract malformations.
4. Requirement non-invasive ventilation support, including the use of a high-flow nasal cannula.
5. Enrollment within the first 72 h of life.

Exclusion criteria

1. Requirement of mechanical ventilation.
2. Severe asphyxia.
3. Prior enrollment in other studies.
4. Diagnosis of necrotizing enterocolitis (NEC).
5. Diagnosis of periventricular leukomalacia (PVL).
6. Diagnosis of intracranial hemorrhage grade III or higher.
7. Need for reinitiation of non-invasive ventilation support after the start of the OMIs.

There are termination criteria and they are as follows: (1) Occurrence of severe adverse events and complications during the trial. (2) Withdrawal of consent by the parents of the subject. Patients who enter the trial but fail to complete it due to any reason will be considered dropout cases. This includes cases where the doctor determines that the subject has withdrawn from the trial, as well as cases where the subject withdraws on their own, regardless of the timing or reason for withdrawal. All data on the infants will still be recorded.

### Who will take informed consent? {26a}

The coordinating center will aid in obtaining consent for the research team to approach individuals who may qualify for participation. An eligible status will be determined by a research nurse, and she will also provide information about the study and ensure that signed informed consent is obtained.

### Additional consent provisions for collection and use of participant data and biological specimens {26b}

N/A: There will be no collection of biological specimens as part of this trial.

### Interventions

#### Explanation for the choice of comparators {6b}

The studies demonstrate that oral motor intervention conducted after discontinuing non-invasive ventilation for 48 h is effective [18, 19].

#### Intervention description {11a}

The specific intervention plan is shown in Table 1.

**Table 1:** Oral stimulation program [7]

Structure	Stimulation steps	Purpose	Frequency	Duration
Cheek	1. Place index finger at the base of the nose 2. Compress the tissue, move finger toward the ear, then down and toward the corner of the lip (i.e., C pattern) 3. Repeat for other side	Improve range of motion and strength of cheeks and improve lip seal	4 $\times$ each cheek	2 min
Upper lip	1. Place index finger at the corner of the upper lip 2. Compress the tissue 3. Move the finger away in a circular motion, from the corner toward the center and to the other corner 4. Reverse direction	Improve lip range of motion and seal	4 $\times$	1 min
Lower lip	1. Place index finger at the corner of lower lip 2. Compress the tissue 3. Move the finger away in a circular motion, from the corner toward the center and to the other corner 4. Reverse direction	Improve lip range of motion and seal	4 $\times$	1 min
Upper and lower lip curl	1. Place index finger at center of lip 2. Apply sustained pressure, stretch downward toward the midline 3. Repeat for lower lip—apply sustained pressure, and stretch upward toward the midline	Improve lip strength, range of motion and seal	2 $\times$ each lip	1 min
Upper gum	1. Place finger at the center of the gum, with firm sustained pressure, slowly move toward the back of the mouth 2. Return to the center of the mouth 3. Repeat for opposite side	Improve range of motion of tongue, stimulate swallow, and improve suck	2 $\times$	1 min
Lower gum	1. Place finger at the center of the gum, with firm sustained pressure slowly move toward the back of the mouth 2. Return to the center of the mouth 3. Repeat for opposite side	Improve range of motion of tongue, stimulate swallow, and improve suck	2 $\times$	1 min
Internal cheek	1. Place finger at inner corner of lips 2. Compress the tissue, move back toward the molars, and return to corner of lip 3. Repeat for other side	Improve cheek range of motion and lip seal	2 $\times$	2 min
Lateral borders of the tongue	1. Place finger at the level of the molar between the side blade of the tongue and the lower gum 2. Move the finger toward midline, pushing the tongue towards the opposite direction 3. Immediately move the finger all the way into the cheek, stretching it	Improve tongue range of motion and strength	2 $\times$	1 min
Midblade of the tongue	1. Place index at the center of the mouth 2. Give sustained pressure into the hard palate for 3 s 3. Move the finger down to contact the center blade of the tongue 4. Displace the tongue downward with a firm pressure 5. Immediately move the finger to contact the center of the mouth at the hard palate	Improve tongue range of motion and strength, stimulate swallow, and improve suck	4 $\times$	1 min
Elicit a suck	1. Place finger at the midline, center of the palate, and gently stroke the palate to elicit a suck	Improve suck and soft palate activation	N/A	1 min
Pacifier	1. Place pacifier in mouth	Improve suck and soft palate activation	N/A	3 min

##### Early OMIs group

On the basis of routine care, we will implement an oral motor intervention program developed by American researcher Fucile, adapted for the Chinese context made by the domestic expert, Lv et al. [7, 15]. The program includes oral stimulation, which involves stimulation of the lips, cheeks, gums, and tongue, along with non-nutritive sucking. The intervention will start once the infant begins to receive milk and exhibits stable vital signs. It will be conducted once a day, for 15 min at a time, and will continue until the infant reaches full oral feeding and maintains it for 48 h without the need for tube feeding. The timing of each intervention is set for 15–30 min before any feeding session, within the hour of 8:00–18:00. All interventions will be performed by charge nurses who have received uniform training. If the infant experiences changes in their condition such as decreased oxygen saturation, bradycardia, or asphyxia, the intervention will be stopped.

##### Late OMIs group

The interventions for OMIs will be initiated 48 h following the discontinuation of nasal continuous positive airway pressure. The early OMIs group and the late OMIs are the same in every aspect, with the sole distinction being the difference in the start time.

#### Criteria for discontinuing or modifying allocated interventions {11b}

Modifying the allocation intervention is expected to be unlikely. Nevertheless, parents have the option to remove their preterm infant from the study at any time for any reason.

#### Strategies to improve adherence to interventions {11c}

Every possible endeavor will be undertaken to maintain the authenticity of the intervention. Before the study begins, individuals participating in the research will receive standardized training in accordance with the research manual. Only after passing the assessment will they be allowed to join the study. Any divergence from the protocol will be meticulously recorded, with an intervention fidelity checklist being completed by the research nurse at the conclusion of the procedure.

#### Relevant concomitant care permitted or prohibited during the trial {11d}

To ensure the accuracy and comparability of trial results, during the study, other unrelated oral motor rehabilitation treatments will be prohibited.

#### Provisions for post-trial care {30}

Patients and their families will receive free consultation and follow-up during and after the research.

#### Outcomes {12}

##### Primary outcome

Transition time is defined as the number of days needed to progress from the initiation of feeding to the establishment of full oral feeding. In this study, full oral feeding is defined as achieving a feeding volume of 120 ml/(kg/day) without the necessity for tube feeding for at least 48 h [20]. The corrected gestational age (PMA) will be recorded both at the initiation of oral feeding and upon the achievement of full oral feeding.

##### Secondary outcomes

**Feeding performance**

This includes feeding efficiency, defined as the average milk intake per minute, and feeding effectiveness, which is the milk intake orally within the first 5 min divided by the ordered milk volume. Feeding performance will be observed at two distinct time points: the day on which full oral feeding is achieved and the day of discharge. Nursing staff who are not involved in the study will record the volume of oral intake and the duration of feeding, from which they will calculate both the feeding efficiency and effectiveness.

**Oral motor function**

The neonatal oral-motor assessment scale (NOMAS) will be used to evaluate the oral motor function of the study subjects. This scale evaluates the jaw and tongue function of newborns across three dimensions: normal sucking, disorganized sucking, and sucking disorders. Evaluations will be performed at baseline, weekly, upon achievement of full oral feeding, and at the time of discharge. The assessment is scheduled to take place daily between 9 a.m. and 10 a.m. During each assessment, the evaluator will also be required to record a 2-min assessment video for documentation and further analysis purposes.

**Weight gain**

Weight gain speed will be calculated as follows: [weight gain rate (g/kg /day)] = [1000 × ln(discharge weight/birth weight)]/(age at discharge  − age at return to birth weight).

**Length of hospital stay**

The length of hospital stay will be determined by recording the admission and discharge dates for each infant, allowing for calculation of the total duration of hospitalization.

**Height and head circumference**

Height and head circumference will be recorded both at admission and discharge.

**Follow-up**

The feeding performance, height, weight, head circumference will be evaluated at the corrected ages of 6 and 12 months and neurodevelopmental outcomes will be evaluated at the corrected ages of 18 to 22 months.

**Safety indicators**

Safety indicators in this study encompass the rate of adverse events occurring during the intervention period. Adverse events are defined as incidents related to this study that are unplanned, unexpected, or typically undesirable such as emesis, aspiration, oxygen desaturation, bradycardia, and other non-routine nursing incidents that may compromise child safety or elicit nursing complaints. Additionally, these incidents may also encompass asphyxiation, tachycardia, tachypnea, bradypnea, apnea, and oral infections.

#### Participant timeline {13}

The timeline is presented in Table 2.

**Table 2:**
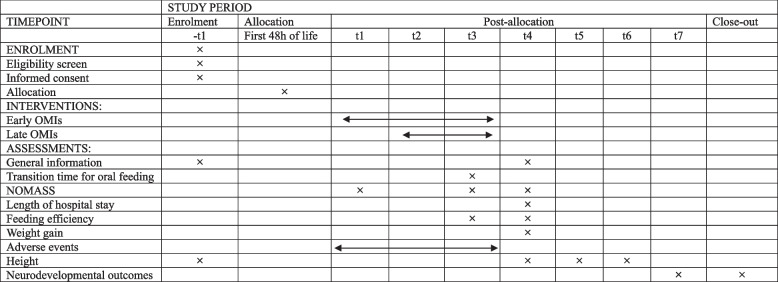
Study timeline

t1 started to feed; t2 after 48 h of discontinuing non-invasive ventilation; t3 full oral feeding; t4 discharge; t5 follow-up at 6 months after discharge; t6 follow-up at 12 months after discharge; t7 at the corrected age of 18–22 months

#### Sample size {14}

The sample size was calculated using the online calculator available at http://powerandsamplesize.com/, based on the primary outcome measure of transition time—the duration time from the initiation of feeding to achieving full oral feeding. A two-sample independent means comparison method was applied for this calculation. The sample size calculation accounts are based on a type I error probability of 5% and a statistical power of 80%. According to our pilot study (*n* = 26), the mean transition times for the two groups were found to be μ1 = 11.36 days for the early OMIs group, μ2 = 13.40 days for the late OMIs group, with a *σ* = 4.99 days. The two groups were allocated in a 1:1 ratio; the initial calculation indicated a need for 126 participants per group. To account for potential dropouts or exclusions, the sample size was augmented by 10%, resulting in a total of 280 participants, or 140 participants per group.

#### Recruitment {15}

Recruitment is currently in progress. We estimate an average enrollment of 12 participants per month based on the anticipated number of preterm admitted to the NICU and recruitment rates observed in our previous pilot studies. The research team will maintain daily checks of the electronic medical record system and communicate with the clinical team to identify infants who potentially meet the eligibility criteria, along with their parents. The research nurse will approach the eligible infants' parents, provide them with information, and obtain their consent.

## Assignment of interventions: allocation

### Sequence generation {16a}

We employ the method of generating random numbers using the web page ( https://www.random.org/) through a sequence generator for the purpose of random allocation. The 280 eligible study subjects who meet the inclusion criteria will be sequentially assigned numbers ranging from 1 to 280, corresponding to their order of admission. Each number assigned to a participant corresponds to a unique random number generated by the website. Based on the outcome of this random number generation, participants assigned odd numbers will be placed in the early OMIs group, whereas those with even numbers will be categorized into the late OMIs group.

### Concealment mechanism {16b}

The randomization scheme will be concealed by placing the random allocation sequences in opaque envelopes. Each envelope corresponded to a sequential number (aligned with the admission order). The management of the randomization scheme was independently handled by a research nurse not involved in the intervention. After obtaining informed consent from the participants, they will be allowed to open the envelopes to reveal their assigned group upon which the grouping result was obtained.

### Implementation {16c}

After obtaining consent and before proceeding with the medically necessary procedure, the research nurse will perform randomization of the preterm to determine the allocation of the study intervention.

## Assignment of interventions: blinding

### Who will be blinded {17a}

Blinding will be implemented for study participants, their families, data collectors, and statisticians, meaning that neither the subjects nor their families, data collectors, and statisticians are aware of the group assignments in the trial.

### Procedure for unblinding if needed {17b}

We do not expect the need to reveal the data’s allocation status before conducting the analyses.

## Data collection and management

### Plans for assessment and collection of outcomes {18a}

The measurement of baseline data takes approximately 15 min, NOMAS assessment requires 2 to 4 min, measuring height and head circumference takes 3 to 5 min, and the total data collection time is approximately 30 min. Premature infants will be followed up until completing neurodevelopmental assessment at 18–22 months of age.

### Plans to promote participant retention and complete follow-up {18b}

Free consultation and follow-up will be provided to patients and their families during the study.

### Data management {19}

Establishment of the data monitoring committee to strictly control the data collection. Demographic and background clinical data describing the sample that will be collected from the electronic medical record system will include maternal parity, maternal age, maternal disease, pregnancy complications and co-morbidities, conception method, delivery method, infant GA at birth, infant birth weight, infant height, infant head circumference, infant Apgar scores, infant sex, diagnoses, and so on.

### Confidentiality {27}

The identities of study participants will remain confidential and will not be disclosed in any research reports or publications. All study participant data and videotapes will be securely stored within a locked metal cabinet at the NICU. To further protect privacy, participant names will be completely removed and replaced with a unique code for identification purposes. This code will be securely stored within a locked metal cabinet in the same place. All research records will be retained for a minimum period of 10 years and will only be accessible to authorized research staff.

### Plans for collection, laboratory evaluation, and storage of biological specimens for genetic or molecular analysis in this trial/future use {33}

N/A: There will be no collection of biological specimens as part of this trial.

## Statistical methods

### Statistical methods for primary and secondary outcomes {20a}

The intention-to-treat analysis (ITT) will be the main analysis, with a per-protocol set (PPs) analysis conducted simultaneously. A significance level of *P* < 0.05 will be considered statistically significant. In the ITT analysis, all subjects will be included in the group to which they are assigned, regardless of whether they completely received the intervention. (1) Descriptive statistics will be used to summarize baseline characteristics. Continuous variables will be presented as Mean ± Standard deviation and categorical variables as percentages (%). (2) For comparisons between groups, Student *t*-tests will be applied for continuous variables, and chi-square tests will be utilized for categorical variables. (3) Assessment of efficacy: for continuous variables, independent Student *t*-tests will be employed for evaluation if conditions are met. (4) For data that do not follow a normal distribution, ordinal data, or data with significantly unequal variances, non-parametric tests will be used. The statistical analysis will be conducted using the medical statistical software SPSS 23.0 (SPSS, IBM Corporation, Armonk, NY, USA).

### Interim analyses {21b}

There are no intentions for an interim analysis. This decision is grounded in three key reasons: first, the OMIs is extensively utilized and has demonstrated a reliable safety record, leading us to conclude that interim analyses to affirm safety may be unnecessary. Second, our study employs a blinded design, eliminating the anticipation of disclosing data allocation status prior to analysis, where interim analyses could potentially compromise the design’s integrity. Last, from a statistical perspective, interim analyses carry the risk of generating false positives. In the absence of adequate statistical adjustments, regular examination of the data could yield misleading insights.

### Methods for additional analyses (e.g., subgroup analyses) {20b}

The sub-group analysis based on gestational age will be performed. If required, according to the type of variable and their distributions, linear or logistic regressions will be performed. Multivariate regressions will also be conducted for selected outcomes, if necessary. In cases where a baseline characteristic in the enrolled population differs between the two groups with a *P* < 0.05 in the univariate analysis, then the results will be adjusted for that variable).

### Methods in analysis to handle protocol non-adherence and any statistical methods to handle missing data {20c}

We anticipate minimal protocol non-adherence issues, given that data collection takes place during a single intervention involving infant participants.

### Plans to give access to the full protocol, participant level-data, and statistical code {31c}

This protocol will be made accessible through a publication.

## Oversight and monitoring

### Composition of the coordinating center and trial steering committee {5d}

The coordinating center, staffed by two head nurses and one assistant head nurse, will play a crucial role in ensuring smooth coordination and communication across the various research teams, distributing tasks, and updating all relevant departments on any changes to the research protocol. Additionally, this center will spearhead the organization of biannually meetings that include research members and other groups, while assisting research nurses in securing informed consent.

The trial steering committee, featuring two doctors and a nurse, will provide essential guidance on the design of the research, address any challenges that arise during its execution, and supervise the quality control team’s activities to uphold the highest standards of research quality and integrity.

### Composition of the data monitoring committee, its role, and reporting structure {21a}

A data safety monitoring committee will be instituted, comprised of three professionals with specialized knowledge in neonatology and nursing. These committee members will be maintained independent from the investigators, ensuring there are no scientific, financial, or other conflicts of interest with the study. Furthermore, the committee is tasked with safeguarding the authenticity and integrity of the data, ensuring that the study’s findings are reliable and credible.

### Adverse event reporting and harms {22}

Any adverse events associated with this study that occur will be documented and reported. Infants will be under constant monitoring during the intervention, including continuous oxygen saturation and cardiorespiratory recording) to mitigate any potential risk of OMIs.

#### Risk 1：Vomiting and Aspiration

 Prevention: Select optimal times when the infant is calm, not immediately before feeding,  and fully awake. Management: if vomiting occurs, cease the procedure immediately.

Place the infant in a prone position with their face down, gently patting their back to facilitate the flow of vomit. After vomiting, position the infant on their side to prevent aspiration pneumonia. In case of aspiration, follow the emergency protocol for choking and aspiration established by the Neonatology Department of West China Second University Hospital, Sichuan University, to address the situation effectively.

#### Risk 2: Infection

Prevention: adhere rigorously to hand hygiene and disinfection isolation protocols. Thoroughly apply the “seven-step hand-washing method” before any contact with the infant and wear sterile gloves conducting any procedure. Management: administer appropriate medications for treatment based on the identified pathogen, as needed.

### Frequency and plans for auditing trial conduct {23}

The trial steering committee, the quality control team alongside the data monitoring and ethics committee will convene biannually to scrutinize several key areas: fidelity to the research trial’s protocol, the data’s accuracy and integrity, adherence to ethical guidelines, effective execution of safety reporting and monitoring protocols for participants, swift recognition and resolution of potential safety concerns, and the trial’s compliance with applicable national and international regulations, guidelines, and standards.

### Plans for communicating important protocol amendments to relevant parties (e.g., trial participants, ethical committees) {25}

If there is any change to the protocol, we will proceed with the following steps: (1) first, the funder will be informed; (2) second, investigators and the ethical committee will receive a copy of the amended protocol; (3) third, any deviations from the protocol will be fully documented using a breach report form; and (4) finally, we will update the protocol in the clinical trial registry.

## Dissemination plans {31a}

The results of study will be placed on targeting NICU staff and researchers. These study reports will be disseminated globally and nationally through influential academic peer-reviewed journals.

### Patient public involvement

We have engaged the parents of the patients to share their insights on the research protocol. Furthermore, we have gathered data on the outcome measures they find important, focusing on elements like the possibility of enabling earlier discharge for infants.

## Discussion

Preterm infants often face difficulties in oral feeding, and the development of oral feeding skills is crucial for their growth and survival. OMIs have been shown to be effective in improving the oral feeding performance of preterm infants [21–23]. However, there is no standardized protocol for the initiation time of OMIs, and the long-term effects of OMIs on preterm infants’ growth, development, and neurodevelopmental outcomes are unclear.

This study aims to compare the effects of different initiation times of OMIs on feeding outcomes, growth, development, and neurological outcomes in preterm infants. The primary outcome is transition time. Secondary outcomes include feeding performance, oral motor function, and long-term growth and neurodevelopmental outcomes.

The study design is a single-blind, randomized, and controlled trial. The results of this study may help to establish a standardized protocol for the initiation time of OMIs and provide evidence-based guidance for healthcare workers in the NICU to improve the oral feeding performance of preterm infants. In addition, the study may provide insights into the long-term effects of OMIs on preterm infants’ growth, development, and neurodevelopmental outcomes, which may have significant implications for the quality of survival of preterm infants and the burden of preterm birth on families and society.

## Trial status

The trial is currently in the recruitment phase. Recruitment began on October 20, 2023, and is estimated to be completed on October 20, 2025. Protocol Version 1.0.

## Data Availability

The study team will have access to the final trial.

## Abbreviations

NICU: Neonatal Intensive Care Unit
OMIs: Oral Motor Interventions
NEC: Necrotizing Enterocolitis
PVL: Periventricular Leukomalacia
